# New host associations and a novel species for the gall-inducing acacia rust genus *Ravenelia* in South Africa

**DOI:** 10.3897/mycokeys.43.25090

**Published:** 2018-12-04

**Authors:** Malte Ebinghaus, Wolfgang Maier, Michael J. Wingfield, Dominik Begerow

**Affiliations:** 1 AG Geobotanik, Ruhr-Universität Bochum, Germany Ruhr-Universität Bochum Bochum Germany; 2 Institute for Epidemiology and Pathogen Diagnostics, Julius Kühn-Institut (JKI), Federal Research Centre for Cultivated Plants, Braunschweig, Germany Julius Kühn-Institut Braunschweig Germany; 3 Forestry and Agricultural Biotechnology Institute (FABI), University of Pretoria, South Africa University of Pretoria Pretoria South Africa

**Keywords:** *Raveneliaxanthophloeae* sp. nov., *
Vachellia xanthophloea
*, novel host record, aecial galls, teliospore morphology, intraspecific variability, Principal Component Analysis

## Abstract

Trees in the genus *Vachellia* (previously *Acacia*) are commonly infected by the gall-inducing rusts *Raveneliamacowaniana* and *R.evansii*. Rust galls bearing aecial infections and relating uredinial and telial infections on the leaves of nine *Vachellia* species not previously recorded to be infected by *Ravenelia* spp. have recently been collected in South Africa. The rust fungi causing these infections were characterised using molecular phylogenetic analyses of DNA sequence data of the LSU and ITS rDNA regions as well as morphological examinations. The host range of *R.macowaniana* and *R.evansii* was thus re-assessed and extended from four to nine species and from one to three species, respectively. Application of Principal Component Analyses (PCA) of telial morphological characters provided evidence of an effect of the host species on the teliospore morphology in *R.evansii*, but only minor effects in *R.macowaniana*. A novel gall-inducing *Ravenelia* sp. closely related to *R.macowaniana*, was found on *Vachelliaxanthophloea* and it is described here as *R.xanthophloeae*.

## Introduction

Trees in the genus *Vachellia* (formerly Acaciasubg.Acacia) and referred to here as acacias make up one of the most prominent floral elements of the Southern African landscape. Acacias can be found in all South African biomes. Here they play important ecological roles by providing food for insects, birds and game, as well as improving soil fertility through nitrogen fixation by their associated rhizobia (Ross 1979, Coe and Coe 1987, Coates Palgrave 2005, Smit 2008, Grellier et al. 2012).

In South Africa, acacias are commonly infected by rust fungi (Pucciniales) of the genus *Ravenelia* (Doidge 1950). *Ravenelia* includes more than 200 described species and is amongst the most species-rich genera of rust fungi (Cummins and Hiratsuka 2003). These fungi are obligate parasites of various genera of the legumes (i.e. in sub-families Mimosoideae, Faboideae, Caesalpinioideae) and they are globally distributed in the tropics and sub-tropics (Dietel 1906, Cummins and Hiratsuka 2003). While the aecial stage of several macrocyclic species is known to cause hypertrophied tissues such as galls and witches brooms within host organs (Dietel 1894, Hernandez and Hennen 2003), the multicellular teliospores of *Ravenelia* are amongst the most complex spore forms found in the rusts.

In South Africa, 20 species of *Ravenelia* have been described, the majority of which infect trees of the acacia genera *Senegalia* and *Vachellia* (Doidge 1939, 1950, van Reenen 1995). Four of these including *R.natalensis* Syd., P. Syd. & Pole-Evans, *R.deformans* (Maublanc) Dietel, *R.macowaniana* Pazschke and *R.evansii* Syd. & P. Syd. induce galls on their hosts. Based on the number of deposited specimens in the National Collection of Fungi, South Africa (PREM) and our own field observations, the macrocyclic and gall-inducing *R.macowaniana* and *R.evansii* are likely the most abundant *Ravenelia* species in South Africa. *Raveneliamacowaniana* has been reported from *V.karroo* only, which in turn is the most frequently occurring acacia in this region. *Raveneliaevansii* has been reported from four acacia species including *V.gerrardii* (Benth.) P.J.H. Hurter, *V.rehmanniana* (Schinz) Kyal. & Boatwr. V.robustassp.robusta (Burch.) Kyal. & Boatwr. and V.sieberianavar.woodii (Burtt Davy) Kyal. and Boatwr. (Doidge 1939, 1950). In contrast, *R.deformans* and *R.natalensis*, both reported from *V.karroo* (Hayne) Banfi and Galasso, have only rarely been collected (Doidge 1939, Farr and Rossman 2017).

During several field surveys focused on re-assessing the diversity of *Ravenelia* species in South Africa, we sampled rust infections associated with galls on eight *Vachellia* species. The aim of this study was to identify the collected rust specimens using morphological and phylogenetic analyses. In addition, new host associations and a new species were reported and the influence of the host on teliospore characters was analysed.

## Material and methods

### Specimens examined

Infected *V.borleae*, *V.davyi*, *V.exuvialis*, *V.hebeclada*, *V.natalitia*, *V.permixta*, *V.swazica* and *V.xanthophloea* trees were sampled during several field surveys in South Africa between 2004 and 2015. Leaves bearing uredinial and telial rust sori and short branches having aecial galls were collected and subsequently dried between paper sheets in a plant press. In total, 49 specimens were studied based on morphology and 31 of these could be used for phylogenetic analyses based on DNA-sequence data. Ten of the 49 specimens were either type or voucher specimens collected in the late 19^th^ and early 20^th^ century and used by Doidge for her studies of the southern African *Ravenelia* spp. (Doidge 1927, 1939). These herbarium specimens were used only for morphological comparisons with the newly collected material (Table 1). Three specimens were deposited in KR, all others in PREM.

**Table 1.** List of specimens included in the present study, including host information, collection data and GenBank accession numbers of rDNA sequences.

Voucher	Species name	Host	Origin	Date	Collector	GenBank accession-Nos.	
						ITS	LSU
PREM61208	* Ravenelia evansii *	Vachellia robusta ssp. robusta	South Africa, North-West Province, Groot Marico, River Still Guest Farm	15 Apr 2009	W. Maier	MG945960	MG945992
PREM61209	“ “	“ “	South Africa, KwaZulu-Natal, Lake St. Lucia	18 Mar 2010	M. Ebinghaus	MG945959	MG945991
PREM2211	“ “	“ “	South Africa, Gauteng, Pretoria, The Willows	6 Apr 1912	I. B. Pole Evans	–	–
PREM6807	“ “	“ “	South Africa, KwaZulu-Natal, Verulam	3 Jul 1913	I. B. Pole Evans	–	–
PREM7105	“ “	“ “	South Africa, KwaZulu-Natal, Verulam	3 Jul 1913	I. B. Pole Evans	–	–
KR-M-43649	“ “	“ “	South Africa, KwaZulu-Natal, Mtunzini	20 Mar 2010	M. Ebinghaus	MG945958	MG945990
PREM61225^†^	“ “	Vachellia sieberiana var. woodii	South Africa, Mpumalanga, R40 north of Nelspruit	22 June 2005	W. Maier	–	–
PREM61228	“ “	“ “	South Africa, KwaZulu-Natal; 30°52'S; 30°18'E	24 Nov 2005	A. R. Wood	MG945957	MG945989
PREM61223	“ “	“ “	South Africa, KwaZulu-Natal; 28°50'27"S; 29°26'5.8"E	23 Mar 2010	M. Ebinghaus	MG945956	MG945988
PREM2403	“ “	“ “	South Africa, KwaZulu-Natal, Cramond	3 June 1912	I. B. Pole Evans	–	–
PREM2539	“ “	“ “	South Africa, KwaZulu-Natal, Estcourt	31 Jul 1912	I. B. Pole Evans	–	–
PREM61881	“ “	“ “	South Africa, Mpumalanga; 25°23'41.8”S; 31°05'08.0”E	14. Feb 2015	M. Ebinghaus	MG945955	MG945987
PREM61224^†^	“ “	* Vachellia davyi *	South Africa, Mpumalanga, R40 north of Nelspruit	27 June 2005	W. Maier	–	–
PREM61005	“ “	“ “	South Africa, Mpumalanga; 35 km east of MBombela; 25°34'21.6"S; 31°10'48.1"E	11 Apr 2013	M. Ebinghaus	MG945967	MG945999
PREM61845	“ “	“ “	South Africa, KwaZulu-Natal, near Pongola; 27°19'27.2"S; 31°26'39.6"E	13. Feb 2015	M. Ebinghaus	MG945968	MG946000
PREM61227	“ “	* Vachellia hebeclada *	South Africa, North-West Province, Leeuwfontein Farm	30 Dec 2006	A. E. van Wyk	MG945969	MG946001
PREM61211^†^	“ “	* Vachellia swazica *	South Africa, Mpumalanga; Marloth Park; 25°20'48.2"S; 31°46'45.7"E	9 Apr 2013	M. Ebinghaus	–	–
PREM61212^†^	“ “	“ “	South Africa, Mpumalanga; Marloth Park; 25°20'44.3"S; 31°46'26.2"E	9 Apr 2013	M. Ebinghaus	–	–
PREM61002	“ “	“ “	South Africa, Mpumalanga; Marloth Park; 25°20'43.0"S; 31°46'38.8"E	9 Apr 2013	M. Ebinghaus	MG945966	MG945998
PREM61008	“ “	“ “	South Africa, Mpumalanga; 25 km east of MBombela; 25°30'43.5"S; 31°10'3.3"E	12 Apr 2013	M. Ebinghaus	MG945965	MG945997
PREM61028	“ “	“ “	South Africa, Mpumalanga; Marloth Park; 25°20'44.4"S; 31°46'26.1"E	9 Apr 2013	M. Ebinghaus	MG945964	MG945996
PREM61846	“ “	Vachellia luederitzii var. retinens	South Africa, KwaZulu-Natal; 15 km south of Jozini; 27°30'57.3"S; 32°00'39.1"E	12 Feb 2015	M. Ebinghaus	MG945961	MG945993
PREM61868	“ “	* Vachellia exuvialis *	South Africa, Mpumalanga; Justicia; 24°52'52.6"S; 31°23'40.3"E	17 Feb 2015	M. Ebinghaus	MG945963	MG945995
PREM61876	“ “	“ “	South Africa, Mpumalanga, Belfast; 24°56'08.7"S; 31°21'52.5"E	17 Feb 2015	M. Ebinghaus	MG945962	MG945994
ME384	“ “	* Vachellia borleae *	South Africa, KwaZulu-Natal; 20 km north of Empangeni; 28°41'30.1"S; 31°43'16.9"E	9 Feb 2015	M. Ebinghaus	MG945971	MG946003
PREM61869	“ “	“ “	South Africa, Mpumalanga; Masibekela; 25°51'36.2"S; 31°49'51.8"E	16 Feb 2015	M. Ebinghaus	MG945970	MG946002
PREM61222	* Ravenelia macowaniana *	* Vachellia karroo *	South Africa, Limpopo, Sekhukhune Land, Winterveld Mine	23 June 2005	W. Maier	MG945975	MG946007
PREM61221	“ “	“ “	South Africa, North-West Province, Hartebeespoort Dam	June /Jul 2005	W. Maier	MG945973	MG946004
PREM61210	“ “	“ “	South Africa, Eastern Cape, Haga Haga	Dec 2005	W. Maier	MG945972	MG946004
PREM61220^†^	“ “	“ “	South Africa, unknown	15 May 2006	W. Maier	–	–
KR-M-43406	“ “	“ “	South Africa, Western Cape, Worcester	20 Dec 2004	M.J. Wingfield	MG945974	MG946006
KR-M-43657	“ “	“ “	South Africa, North-West Province; 25°30'08.2"S; 27°21'32.4"E	8 Mar 2015	M. Ebinghaus	MG945976	MG946008
PREM61875	“ “	* Vachellia permixta *	South Africa, Limpopo, Mokopane; 24°08'52.4"S; 29°02'21.9"E	23 Feb 2015	M. Ebinghaus	MG945982	MG946014
PREM61862	“ “	* Vachellia natalitia *	South Africa, Limpopo, Steelport; 24°41'32.3"S; 30°12'32.3"E	19 Feb 2015	M. Ebinghaus	MG945980	MG946012
PREM61218	“ “	“ “	South Africa, Mpumalanga, Nelspruit	10 Jan 2005	W. Maier	MG945979	MG946011
PREM61219	“ “	“ “	South Africa, Mpumalanga, Nelspruit	10 Jan 2005	W. Maier	MG945977	MG946009
PREM61888^†^	“ “	“ “	South Africa, Mpumalanga, Nelspruit	21 June 2005	W. Maier	–	–
PREM 61216	“ “	“ “	South Africa, Eastern Cape, Port St. John	28 Dec 2005	W. Maier	MG945978	MG946010
PREM61226^†^	“ “	“ “	South Africa, Mpumalanga, South of Nelspruit	16 Mar 2010	M. Ebinghaus	–	–
PREM61214	“ “	“ “	South Africa, Mpumalanga, East of Barberton	2 June 2012	M. Ebinghaus	MG945981	MG946013
PREM61215	* Ravenelia xanthophloeae *	* Vachellia xanthophloea *	South Africa, Mpumalanga, Barberton; 25°46'52.5"S; 31°03'10.7"E	3 Jul 2012	M. Ebinghaus	MG945985	MG946017
PREM61213	“ “	“ “	South Africa, KwaZulu-Natal, Mount Moreland; 29°38'21.6"S; 31°05'27.3"E	16 June 2012	M. Ebinghaus	MG945983	MG946015
PREM61213 ^‡^	“ “	“ “	South Africa, KwaZulu-Natal, Mount Moreland; 29°38'21.6"S; 31°05'27.3"E	16 June 2012	M. Ebinghaus	MG945986	–
PREM61000	“ “	“ “	South Africa, Mpumalanga, Komatipoort; 25°26'10.0"S; 31°57'48.6"E	9 Apr 2013	M. Ebinghaus	MG945984	MG946016
PREM1935^†^	* Ravenelia natalensis *	* Vachellia karroo *	South Africa, KwaZulu-Natal, Winkelspruit	29 Nov 1911	I. B. Pole Evans	–	–
PREM2514^†^	“ “	“ “	South Africa, KwaZulu-Natal, Winkelspruit	6 Jul 1912	E. M. Doidge	–	–
PREM2375^†^	* Ravenelia glabra *	* Calpurnia sylvatica *	South Africa, KwaZulu-Natal, Mulden	1 May 1912	P. MacOwan	–	–
PREM10698^†^	“ “	“ “	not known	not known	P. MacOwan	–	–
PREM20727^†^	“ “	“ “	South Africa, Western Cape, Somerset East	1875	P. MacOwan	–	–

^†^Specimens were only morphologically investigated. ^‡^Specimen representing the aecial infection state.

**Table 2.** Measurements of morphological characters of *Raveneliaevansii*, *R.macowaniana* and *R.xanthophloeae* sp. nov., separately performed for each host species. All size measurements are given in μm. ^†^Data taken from original descriptions by Doidge (1927).

*Ravenelia* on host species	Teliospore diameter	Probasidial cell length	Probasidial cell width	Epispore thickness	Ornamentation length	Cell numbers in diam.
*** R. evansii ***						
* V. robusta * ^†^	50–80	25–30	17–23	4–6	4–6	4–6
* V. borleae *	(52)72–83(92)	(21)23–28(40)	(16)22–26(34)	(2.5)3–4.5(6.0)	(3)3.5–4.5(6)	3–6
* V. davyi *	(83)95–105(115)	1(9)25–30(34)	(12)19–24(32)	(2.5)4–5(6.5)	(2.0)3.5–5(7)	5–7
* V. exuvialis *	(47)70–85(101)	(19)23–28(33)	(15)21–26(30)	(2.5)3.5–4.5(6)	(3)4.5–6(7.5)	3–7
* V. luederitzii *	(79)94–100(115)	(18)23–30(36)	(15)19–25(32)	(2.5)3–5(7)	(1.5)3–5(6)	5–7
* V. robusta *	(81)90–110(118)	(20)22–29(35)	(14)19–25(33)	(3)3.5–5(6.5)	(1.5)3.5–6(8)	5–8
* V. sieberiana *	(63)85–97(112)	(19)23–29(35)	(15)21–24(33)	(2.5)3.5–4.5(6)	(2.5)4.5–6.5(8)	4–8
* V. swazica *	(71)85–105(124)	(16)22–29(39)	(12)18–27(32)	(2)3–5(7)	(2.5)3.5–6(7)	5–8
*** R. macowaniana ***						
* V. karroo * ^†^	60–130	up to 45	18–28	not stated	–	4–7
* V. karroo *	(75)82–105(118)	(21)23–31(35)	(14)17–24(29)	(2)3–5.5(6.5)	–	4–7
* V. natalitia *	(50)80–105(114)	(19)24–34(45)	(14)19–26(34)	(2)3–4.5(5.5)	–	3–6
*** R. xanthophloeae ***						
* V. xanthophloea *	(40)65–75(84)	(19)22–28(40)	(12)18–25(29)	(2)3–4(6)	(0.5)1–2(3)	3–6

### DNA extraction and PCR

Spores from individual sori were collected separately using sterile insect needles. Genomic DNA extractions were made using the INNUPrep Plant DNA Kit (Analytik Jena, Germany) following the manufacturer’s protocols with the following modifications: Spores were crushed using a Retsch mixer mill MM2000 (Retsch, Haan, Germany) by shaking them together with 2 steel beads of 2.5 mm diameter in a 2.0 ml Eppendorf tube. This process was repeated in three consecutive cycles. In the first step, the closed tubes were cooled in liquid nitrogen and immediately shaken for 2 min at 100 Hz. Thereafter 10–40 μl of lysis buffer was added to the tube to loosen spore remnants from the inner side of the Eppendorf tube lid using a vortex mixer followed by a centrifugation step. Samples were again cooled in liquid nitrogen and shaken for an additional 2 min at 100 Hz followed by centrifugation for 1 min at 6000 rcf. The last two steps were repeated once.

For PCR of the ribosomal nrITS and LSU rDNA gene regions, the Taq-DNA-Polymerase Mix (PeqLab, Erlangen, Germany) was used with the primers ITS1F (Gardes and Bruns 1993) and RustITS1F (Toome and Aime 2014), respectively and ITS4BR (Vialle et al. 2009). PCR of the LSU rDNA region was performed using the primer pairs LR0R and LR6 (Vilgalys and Heester 1990). Two additional primers (5.8SrustF: 5’- CGA TGA AGA ACA CAG TGA AAT GTG; D1D2RustR: 5’- CTY TGC TAT CCT GAG GGA) were designed with improved specificity for *Ravenelia* and that reduced amplification of ascomycetous non-target organisms. The thermal cycling conditions for primers ITS1F/ITS4BR and RustITS1F/ITS4BR were as follows: 2 min at 96 °C followed by 40 cycles of 20 sec at 96 °C, 40 sec at 50 °C and 50 sec at 72 °C, final extension was for 5 min at 72 °C; for primers LR0R/LR6: 3 min at 96 °C followed by 40 cycles of 30 sec at 95 °C, 40 sec at 49 °C and 1 min at 72 °C, final extension was for 7 min at 72 °C; for primers 5.8SrustF/D1D2rustR: 3 min at 96 °C followed by 40 cycles of 30 sec at 96 °C, 45 sec at 54 °C and 1 min 20 sec at 72 °C, final extension was for 7 min at 72 °C.

PCR products were purified using Sephadex G-50 columns (Sigma-Aldrich, Steinheim, Germany). Where PCR products showed only weak bands on agarose gels, purification was undertaken using the Zymo Research DNA Clean & Concentrator^TM^-5 Kit (Zymo Research GmbH, Freiburg, Germany) following the manufacturer’s protocol. DNA sequencing was carried out in both directions using the same primers as those used for PCR on a 3130XL Genetic Analyzer (Applied Biosystems) at the sequencing service of the Faculty of Chemistry and Biochemistry of the Ruhr University Bochum, Germany.

### Phylogenetic analyses

Sequences were screened against the NCBI GenBank using the BLASTn algorithm (Altschul et al. 1990) to exclude erroneously amplified contaminants from further processing. Forward and reverse strands of the rust sequences were assembled using Sequencher 5.0 software (Gene Codes Corp., Ann Arbor, MI, USA) and, where necessary, manually edited. In total, 32 sequences were used to construct an alignment of the nrITS and LSU rDNA sequence data, respectively, using MAFFT v6.832b (Katoh and Standley 2014) applying the L-INS-i strategy. Maximum likelihood analyses were conducted in RAxMLGUI v.1.3 (Silvestro and Michalak 2012) using RAxML 8.0.26 (Stamatakis 2014) using the general time reversible model of nucleotide substitution (Lanave et al. 1984) with gamma distributed substitution rates. The analyses were run with a rapid bootstrap analysis using 1000 bootstrap replicates. The analyses were first conducted for each dataset separately and topological congruence was checked visually. As no conflict of supported phylogenetic groupings was observed, the final phylogeny was inferred by combining both datasets of the nrITS and LSU rDNA sequences applying the same methodology as for individual datasets.

Parsimony network analyses were performed using TCS v1.21 (Clement et al. 2000) and the same sequence alignments that were used for the phylogenetic analyses. Gaps were deleted from calculations and the default connection limit of 95% was used.

### Light- and electron microscopic investigations

The spores of the dried herbarium specimens (Table 1) were scraped from leaf surfaces and mounted in lactophenol on microscope slides. A minimum of ten teliospores and 30 urediniospores per specimen were examined. Minimum, maximum and mean values are provided in Table 2. The specimens PREM2211 (*R.evansii*), PREM2403 (*R.evansii*), PREM2539 (*R.evansii*), PREM6807 (*R.evansii*), PREM7105 (*R.evansii*), PREM1935 (*R.natalensis*), PREM2514 (*R.natalensis*), PREM2375 (*R.glabra*), PREM10698 (*R.glabra*) and PREM20727 (R.glabra) were examined at the facilities of the ARC-Plant Protection Institute (ARC-PPRI), Roodeplaat, South Africa using a Leica Dialux 22 EB microscope and a ColorView III CCD colour camera. Measurements were made using analySIS LS software (LS Research Software GmbH, Germany). The remaining specimens were studied at the Ruhr University Bochum, Germany, using a Zeiss Axioplan light microscope. Morphological characteristics were measured using Cell^D v. 3.1 imaging software (Olympus Soft Imaging Solutions GmbH, Germany) and Zen2 lite (Blue Edition) V. 2.0.0.0 (Carl Zeiss Microscopy, 2011, Jena, Germany). Photographs were obtained using a Color View microscope camera (Olympus Soft Imaging System, Germany). For detailed investigations of the spore-surface structures, scanning electron microscopy (SEM) was used. For this purpose, infected leaflets from the herbarium specimens were mounted on double-sided adhesive carbon tape on metal stubs and coated with gold in a sputter coater BAL-TEC SCD OSO (Capovani Brothers Inc, USA). Subsequently, the samples were examined using a ZEISS Sigma VP scanning electron microscope.

### Principal Component Analyses (PCA)

For principal component analyses (PCA) the morphological data collected for all examined rust individuals were separated into sub-sets based on preliminary species assignments representing *R.evansii*, *R.macowaniana* and *Ravenelia* sp. (Groups A, B1 and B2). PCA for all subsets was conducted separately using the R-packages plyr and ggplot2 implemented in R (www.R-Project.org). Six characteristics of teliospores providing numeric data were defined and measured: teliospore diameter, probasidial cell length and probasidial cell width, number of cells in diameter, epispore thickness and ornamentation length. Mean values were calculated for the individual teliospore measurements, scaled and missing values were deleted from analyses.

## Results

### Phylogenetic Analyses

Sequence data from the nrITS and LSU rDNA gene regions were obtained for all 31 newly collected specimens. The alignment of the nrITS sequence dataset had a total length of 764 bp with 133 variable sites of which 131 positions were parsimony informative. The aligned sequences of the LSU rDNA dataset had a length of 922 bases and comprised 31 sequences with 45 variable sites and 40 parsimony informative positions. The combination of the nrITS and LSU rDNA datasets resulted in an alignment with a total length of 1686 nucleotides comprising 32 sequences. The sequence alignment and phylogenetic tree of the combined rDNA sequence data set was deposited at TreeBASE (http://purl.org/phylo; submission IDS22307).

**Figure 1.**
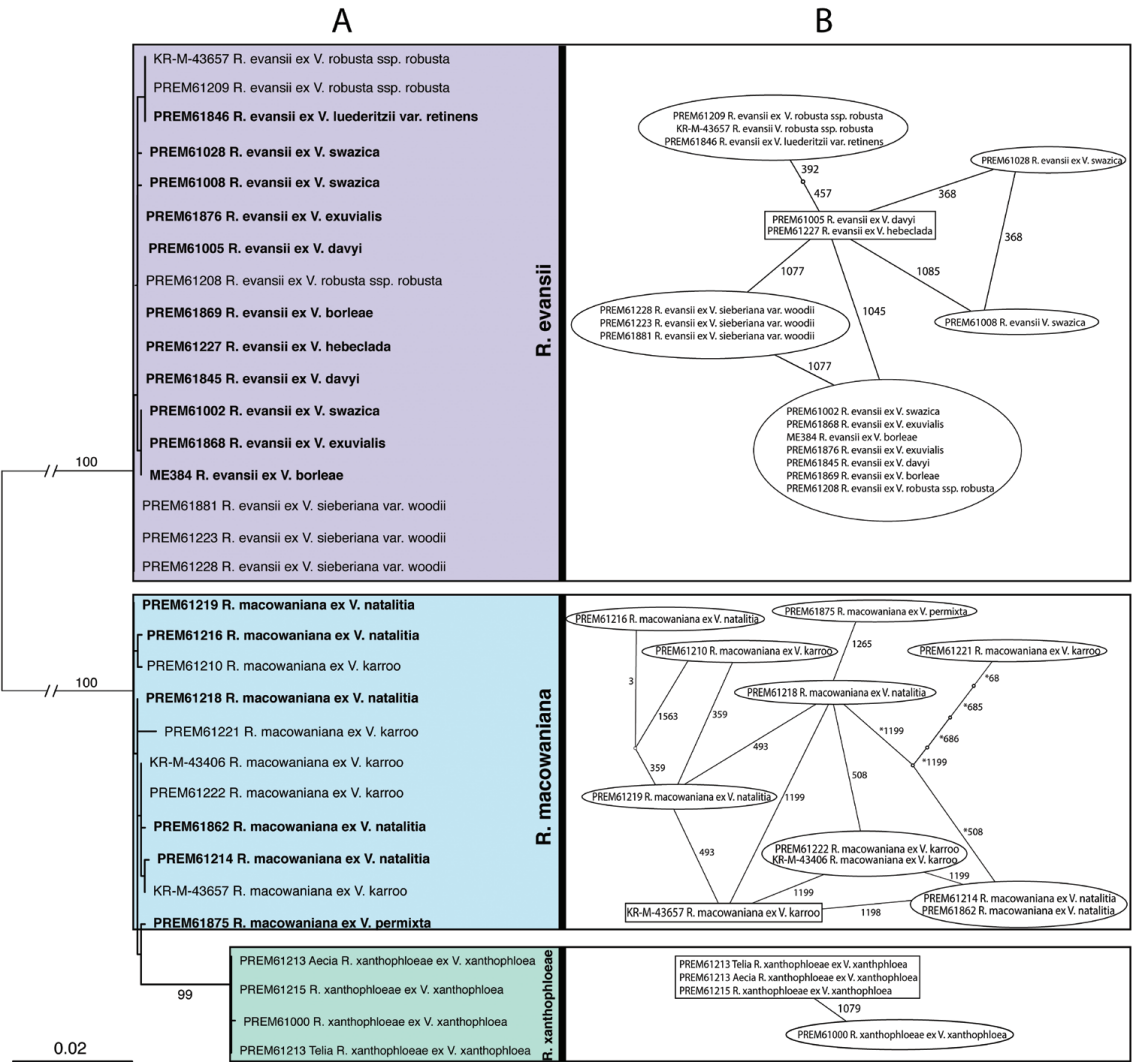
Phylogenetic reconstruction of *Ravenelia* species on different *Vachellia* hosts **A** Maximum likelihood tree with 1000 bootstrap repeats based on combined nrITS and LSU rDNA sequence data. Bootstrap values below 75 are not shown. Three highly supported groups represent *R.evansii*, *R.macowaniana* and *R.xanthophloeae* sp. nov., respectively. Specimens that originated from formerly unreported host species are highlighted in bold **B** Parsimony network analysis based on the same dataset as in the ML-analysis. Each line represents one base substitution while small circles represent intermediate but missing sequences. Numbers next to lines indicate the positions of the substitutions in the alignment. Sequences in rectangular boxes were inferred as ancestral by this analysis.

Maximum likelihood analysis of the combined dataset resulted in a phylogenetic tree that consisted of three highly supported groups representing *R.evansii*, *R.macowaniana* and a novel *Ravenelia* species described below (Fig. 1A).

The phylogenetic group representing *R.evansii* consisted of 17 sequences. Three of these were obtained from Vachelliasieberianavar.woodii (PREM61223, PREM61228, PREM61881) and three from V.robustassp.robusta (KR-M-43649, PREM61208, PREM61209). These are tree species that had previously been reported as hosts of *R.evansii*. Rust specimens collected from the following five *Vachellia* species also clustered in this group: *V.borleae* (ME384 and PREM61869), *V.davyi* (PREM61005), *V.exuvialis* (PREM61868, PREM61876), *V.hebeclada* (PREM61227) and *V.swazica* (PREM61002, PREM61008, PREM61028). These are all newly reported hosts for *R.evansii*.

A second group included the sequences of eleven specimens, five of which originated from *V.karroo* and were identified as *R.macowaniana* (KR-M-43657, PREM61222, PREM61221, PREM61210, KR-M-43406). Five specimens were collected from *V.natalitia* (PREM61214, PREM61862, PREM61218, PREM61219, PREM61216) and one originating from *V.permixta* (PREM61875) also clustered in this group. The latter two hosts are newly reported for *R.macowaniana*.

A distinct clade, nested within the *R.macowaniana* group, was represented by three *Ravenelia* specimens that were isolated from *V.xanthophloea* (PREM61215, PREM61213, PREM61000) suggesting that it represents a novel taxon. For PREM61213, two identical sequences were obtained, one derived from aeciospores and one from teliospores.

The parsimony network analysis, based on the combined set of nrITS and LSU rDNA sequence data, separated three distinct groups each comprising the same specimens representing *R.evansii*, *R.macowaniana* and the novel *Ravenelia* species in our phylogenetic analysis, respectively (Fig. 1B). Network analysis relying on LSU alone could not separate *R.macowaniana* from the novel taxon, while separation of these two groups was observed based on nrITS alone (not shown). The *R.evansii* group consisted of six haplotypes of 17 sequences that differed by a maximum of two substitutions from the inferred ancestral sequences (PREM61005 and PREM61227). In *R.macowaniana*, sequence divergence was more pronounced and consisted of nine haplotypes in a total of eleven sequences. The highest rate of six substitutions was observed for specimen PREM61221 relative to the inferred ancestral sequence (KR-M-43657). Specimens collected on *V.xanthophloea* had only one substitution.

### Morphological analyses

***Ravenelia evansii***

The teliospore morphology of *R.evansii* specimens showed a considerable overall variability in all six investigated teliospore characteristics (Suppl. material 1: Fig. S1, Table 2). The voucher specimens PREM61869 (on *V.borleae*), PREM61876 and PREM61868 (both on *V.exuvialis*) had significantly smaller teliospores compared to the remaining specimens, but variation in this trait could also be observed within single host associations, e.g. within those from *V.davyi* and *V.robusta* (Suppl. material 1: Fig. S1).

**Figure 2.**
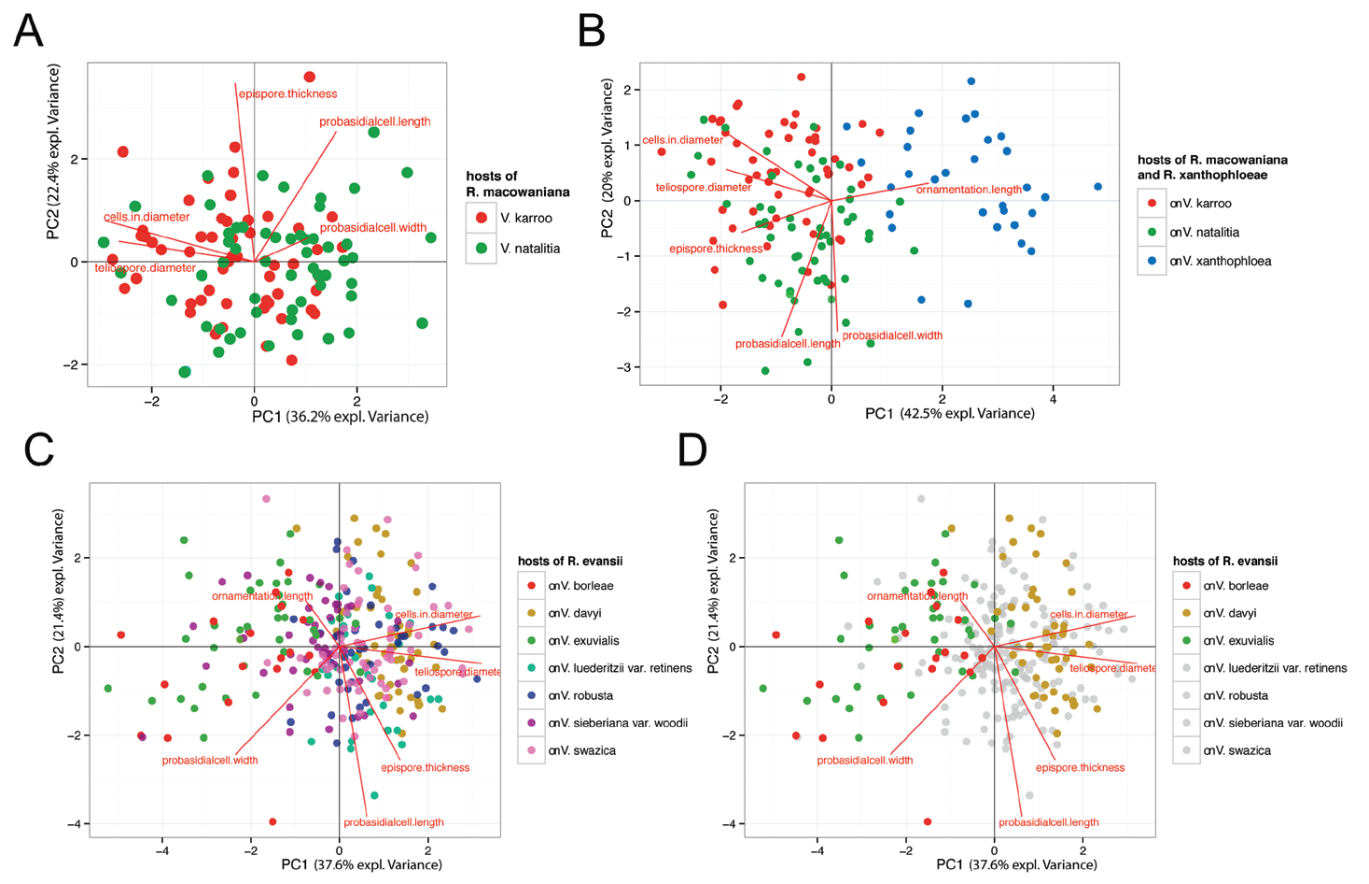
Biplots of a principal component analysis (PCA) of six teliospore characteristics of specimens of **A***Raveneliamacowaniana* originating from *Vachelliakarroo* (red) and *V.natalitia* (green) and **B** in comparison with *R.xanthophloeae* sp. nov. collected from *V.xanthophloea* (blue) **C, D** represent *R.evansii* originating from seven distinct *Vachellia* species. Each dot represents an individual teliospore for which mean values of multiple measurements of all six defined morphological characteristics were calculated. Each colour represents the host species of the individual rust specimen. In **D** only spore representatives collected from *V.borleae*, *V.exuvialis* and *V.davyi* were highlighted to gain better visibility.

The principal component analysis (PCA) of teliospore characteristics clustered several individuals derived from specific hosts into distinct groups (Fig. 2C, D). Individuals that originated from *V.borleae* and *V.exuvialis* clustered more closely and could be separated from those individuals that were collected from *V.davyi* (Fig. 2D). The separation of these individuals was supported by PC1 which could explain 37.6% of the overall variability. The traits ‘cells in diameter’ and ‘teliospore diameter’ were characteristics that corresponded best with this axis (Fig. 2C). These results indicated, that the latter two characters were the most variable traits to separate these individuals with different host associations. However, individuals from *V.luederitzii* var. *retinens, V.robusta* var. robusta, V.sieberianavar.woodii and *V.swazica* showed only weak separation, i.e. less clear patterns of morphological separation.

***Ravenelia macowaniana***

Specimens representing *R.macowaniana* were morphologically more homogeneous compared to *R.evansii*. Here, only spore characteristics such as ‘probasidial cell width’ and ‘epispore thickness’ were often significantly different between investigated specimens (Suppl. material 2: Fig. S2). There was little variation for the characters ‘teliospore diameter’, ‘probasidial cell length’ and the number of ‘cells in diameter’. These characters varied distinctly only between single specimens, e.g. PREM61226 originating from *V.natalitia* had significantly larger teliospores in comparison to specimen PREM61888 that was also collected from this host (Suppl. material 2: Fig. S2).

For the specimens of *R.macowaniana*, PC1 and PC2 could explain 36.2% and 22.4% of the similarity, respectively. However, unlike in *R.evansii*, single teliospore characteristics did not differ significantly in terms of host association (Fig. 2A).

***Ravenelia* sp. nov.**

**Figure 3.**
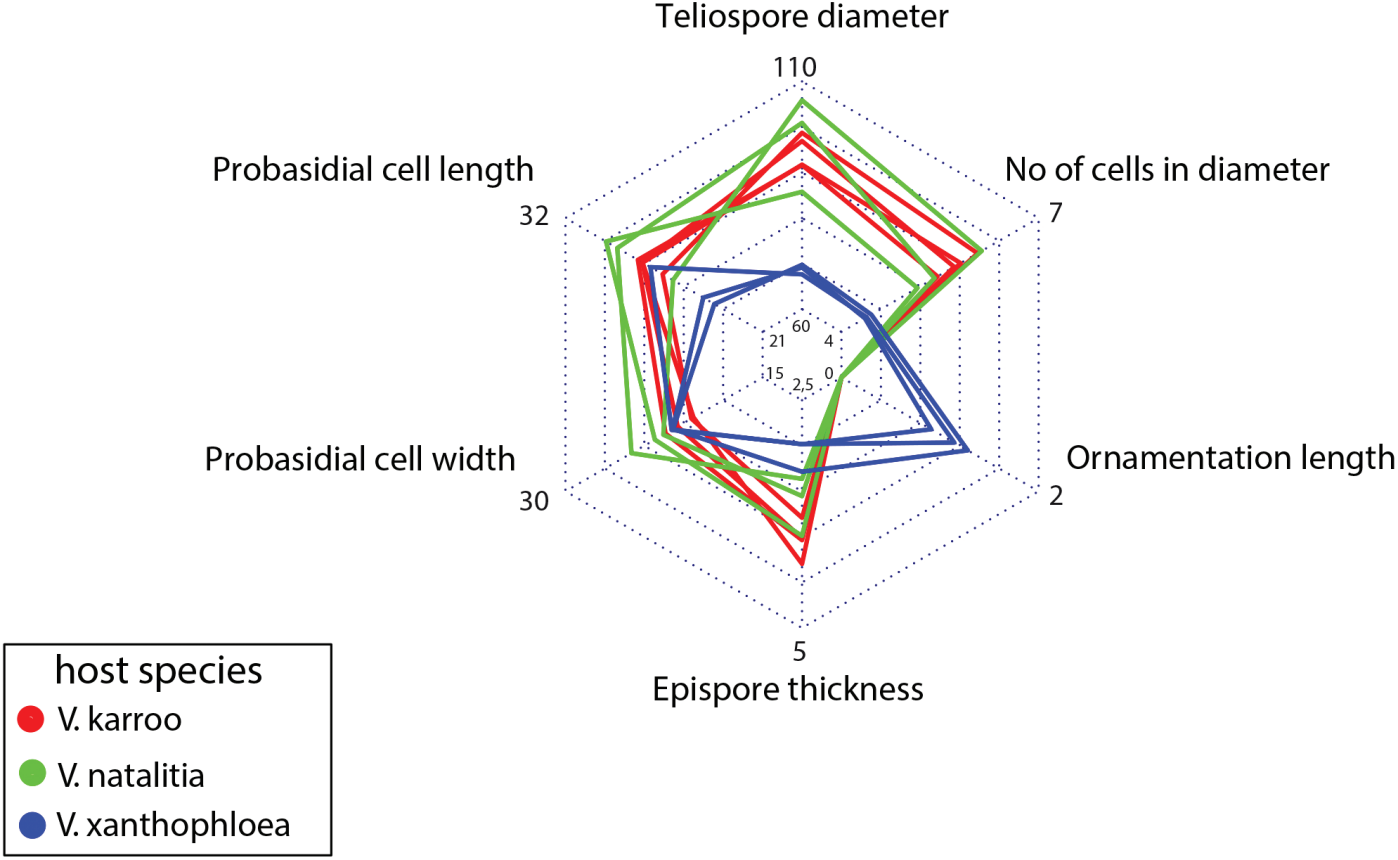
Radarchart of mean values of the morphological investigations of teliospore characteristics of *Raveneliamacowaniana* originated from *Vachelliakarroo* (red), *V.natalitia* (green) and *R.xanthophloeae* on *V.xanthophloea* (blue). Numbers on y-axis represent the respective minimum and maximum values. This radarchart reveals the morphological differences between *R.macowaniana* and *R.xanthophloeae*.

Due to similar teliospore characteristics, *R.macowaniana* was compared using PCA to individuals of the undescribed *Ravenelia* species collected on *V.xanthophloea* in order to characterise and, if possible, to contrast both morphologies. The PCA separated two groups that corresponded well with *R.macowaniana* and the novel *Ravenelia* species and showed very little overlap in morphological characteristics (Fig. 2B). Separation between both species groups was mostly seen in PC1 that could explain 42.5% of the overall variability with the ‘ornamentation length’ corresponding best to this axis (Fig. 2B). Although less distinct in the PCA, mean values of teliospore characters also revealed that the characters ‘epispore thickness’, ‘probasidial cell length’ and especially the ‘teliospore diameter’ and ‘cells in diameter’ are valuable characters for the discrimination of both species (Fig. 3). All spore measurements, derived from the six defined spore characteristics that were used for PCA, are available as an excel-file in the Suppl. materials 3, 4: tables S1, S2: ‘PCA-table *R.evansii*’ and ‘PCA-table *R.macowaniana*’, respectively.

### Taxonomy

#### Ravenelia xanthophloeae

Taxon classificationFungiPuccinialesRaveneliaceae

Ebinghaus, W. Maier & Begerow sp. nov.

824073

Figure 4A–H

##### Type.

South Africa, KwaZulu-Natal, 29°38'21.6"S; 31°05'27.3"E, on leaves and gall-transformed inflorescences of *Vachelliaxanthophloea* (Benth.) P.J.H. Hurter (Fabaceae: Mimosoideae), 16 June 2012, M. Ebinghaus ME188, (holotype: PREM61213); South Africa, Mpumalanga, 25°46'52.5"S; 31°03'10.7"E, on leaves of *Vachelliaxanthophloea* (Benth.) P.J.H. Hurter (Fabaceae: Mimosoideae), 3 July 2012, M. Ebinghaus ME174, (paratype: PREM61215); South Africa, Mpumalanga, 25°26'10.0"S; 31°57'48.6"E, on leaves of *Vachelliaxanthophloea* (Benth.) P.J.H. Hurter (Fabaceae: Mimosoideae), 9 April 2013, M. Ebinghaus ME248, (paratype: PREM61000)

##### Etymology.

The name refers to the host tree, *Vachelliaxanthophloea*.

##### Description.

**Figure 4.**
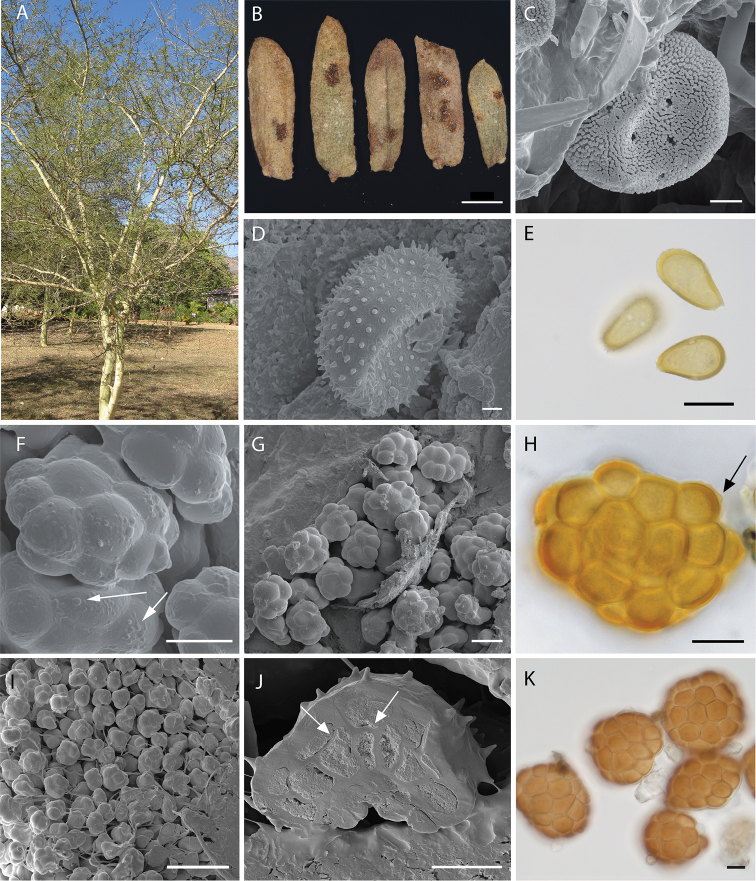
Infected host organs and spore images of *R.xanthophloeae* (**A–H**), *R.natalensis* (**I**), *R.* e*vansii* (**J**) and *R.macowaniana* (**K**) **A** Infected individual of *V.xanthophloea*. Leaves were prematurely shed in comparison with uninfected trees **B** Telia on leaflets of *V.xanthophloea***C** SEM of an aeciospore showing scattered germpores **D** SEM of an urediniospore **E** Urediniospores seen in LM **F** SEM view of a teliospore of *R.xanthophloeae*. The arrows indicate irregularly arranged verrucose ornamentations **G** Telium of *R.xanthophloeae* seen in SEM **H** LM view of a teliospore. The arrow indicates irregularly arranged verrucose ornamentations **I** Teliospores of *R.natalensis* with long pedicels **J** SEM picture of median section of a teliospore of *R.evansii*. Arrows indicate 2-celled probasidial cells **K** LM picture of teliospores of *R.macowaniana*. Scale bars: 1 mm (**B**), 4 μm (**C**), 2 μm (**D**), 20 μm (**E**), 20 μm (**F–H, J–K**), 40 μm (**I**).

Spermogonia not found. Aecia on rust-induced galls, which are formed instead of inflorescences. Aeciospores globose to sub-globose, often angular, yellowish-transparent in light microscopy, light brown when dry (19.0)21.0–24.0(28.5) × (14.5)18.0–20(21.5) μm, cell wall (1.0–)2.0(–3.0) μm thick, densely verrucose, germ pores numerous, scattered (Figure 4C). Peridia and peridial cells could not be described because only disintegrated aecia were present in the dried herbarium material.

Uredinia amphigenous on leaves, but mostly on the abaxial side of the leaflets, scattered or in small groups, minute, 0.1–0.2 mm, erumpent and surrounded by the torn epidermis; ellipsoidal to roundish, light-brown to blackish; paraphyses numerous, scattered within sorus; capitate, thickened end ovoidal, about 19–20 × 11–13 μm, cell wall 2–3 μm, light-brown, smooth; urediniospores ovoidal to broadly ellipsoidal or sometimes subglobose, (18)23–26(38) × (13)16–20(25) μm, spore wall evenly 1.5–2.0 μm thick with densely echinulate aculei (Figure 4D), germ pores 5–6, subequatorial to equatorial (Figure 4E).

Telia replacing the uredinia; teliospores often irregularly shaped from top view; single probasidial cells distinctly arched upwards (Figure 4F–H), orange-brown to pale brown, teliospore surface in general smooth but with single and irregularly arranged small verrucae, (Figure 4F–I); teliospores varying in size from (40)65–75(84) μm in diameter with mostly 4–5 probasidial cells in a cross-section, rarely 3 or 6 cells; central cells in two layers, 32–40 μm in lateral view; marginal cells in a single layer, (19)22–28(33) μm in lateral view and (12)18–25(29) μm from above; upper cellwall (2)3–4(6) μm thick; verrucose ornamentations (0.5)1–2(3) μm in height (Figure 4F, H), rarely with protuberances of up to 7 μm in height; cysts smooth and hyaline, of variable number but often appear in same number as marginal probasidial cells, swelling in water but only slightly in lactophenol, pedicels compound, not persisting.

##### Notes.

In South Africa, *R.macowaniana*, *R.glabra* Kalchbr. & Cooke and *R.deformans* (Maublanc) Dietel are the only known species that exhibit two-layered probasidial cells and smooth teliospores. While the first character is shared by *R.xanthophloeae*, the teliospore surface bears small and irregularly arranged small warts clearly visible in SEM (Fig. 4F). However, these can easily be overlooked in light microscopy (Fig. 4H) and could potentially lead to misidentification. Specifically, *R.macowaniana* differs from *R.xanthophloeae* in the overall size of its teliospores (Table 2; Figure 4K) and the urediniospores have four equatorial germ pores whereas those of *R.xanthophloeae* have five to six equatorial germ pores. The teliospores of the microcyclic *R.glabra* Kalchbr. & Cooke are about twice the size (120–160 μm) of those of *R.xanthophloeae* and its oblong urediniospores are significantly larger (32–48 × 14–21 μm). This rust has also been reported only from *Calpurniasylvatica* (Burch.) E. Mey (Fabaceae) (Doidge 1927). The demicyclic *R.deformans* (Maublanc) Dietel was synonymised with the neotropical *R.hieronymi* Speg. based on nearly identical morphology and congruent life cycle characteristics (Hernandez and Hennen 2003; Hennen et al. 2005) but conspecificity of these two rust fungi is doubtful as they infect distinct host species and occur each on different continents. However, both species produce aecia that induce malformations in young branches, which is a characteristic similar to the newly described *R.xanthophloeae*. With a size of 60–120 µm and 75–120 µm, respectively, the teliospores of *R.deformans* and *R.hieronymi* are, however, on average significantly larger and develop intermingled with the aecia (Doidge 1927), while *R.xanthophloeae* is macrocyclic and aecia, uredinia and telia are produced in spatially separated sori.

The teliospores of *R.xanthophloeae* may also be confused with those of *R.natalensis* Syd., P. Syd & Pole-Evans, but they are significantly smaller in size (30–50 μm diam.) and possess extraordinarily long and persistent pedicels (up to 110 μm; Sydow 1912, Fig. 4I). In *R.natalensis*, the aparaphysate uredinia and telia are confluent and cover large areas on the branches of the host (Sydow and Sydow 1912; own observations), while the specimens of *R.xanthophloeae* examined in this study have minute uredinia with numerous paraphyses and telia not exceeding 200 μm in diameter.

##### New host records.

Morphological and molecular phylogenetic analyses based on nrITS and nrLSU data confirmed new host records for *R.macowaniana* and *R.evansii* that will be reported in the following section. An emended species decription for *R.evansii* is also provided.

#### Ravenelia macowaniana

Taxon classificationFungiPuccinialesRaveneliaceae

Pazschke & Hedwigia, 23: 30 and 59 (1894)

Figure 4K

Ravenelia macowaniana Pazschke & Hedwigia, 23: 30 and 59 (1894). On leaves and in gall-transformed inflorescences of Vachellianatalitia (E.Mey) Kyal. & Boatwr. and on leaves of V.permixta (Burtt Davy) Kyal. & Boatwr. (Fabaceae: Mimosoideae).

##### Specimens examined.

South Africa, Limpopo, Steelport, 24°41'32.3"S; 30°12'32.3"E, on leaves of *Vachelliapermixta*, 23 February 2015, M. Ebinghaus ME424 (PREM61875); South Africa, Limpopo, Steelport, 24°41'32.3"S; 30°12'32.3"E, on leaves of *V.natalitia*, 19 February 2015, M. Ebinghaus ME416 (PREM61862); South Africa, Mpumalanga, East of Barberton, on leaves of *V.natalitia*, 2 June 2012, M. Ebinghaus ME158 (PREM61214); South Africa, Mpumalanga, Nelspruit, on leaves of *V.natalitia*, 10 January 2005, W. Maier WM3292 (PREM61218) and WM3294 (PREM61219); South Africa, Mpumalanga, South of Nelspruit, on leaves of *V.natalitia*, 10 March 2010, W. Maier WM3590 (PREM61226); South Africa, Mpumalanga, Nelspruit, on leaves of *V.natalitia*, 21 June 2005, W. Maier WM3423 (PREM61888); South Africa, Eastern Cape, Port St. Johns, on leaves of *V.natalitia*, 28 December 2005, W. Maier WM3453 (PREM61216); South Africa , Limpopo, Sekhukhune Land, Winterveld Mine, on leaves of *V.karroo* 23 June 2005, W. Maier WM3433 (PREM61222); South Africa, North-West Province, Hartebeesspoort Dam, on leaves of *V.karroo* June/July 2005, W. Maier WM3448 (PREM61221); South Africa, Eastern Cape, Haga Haga, on leaves of *V.karroo*, December 2004, W. Maier WM3485 (PREM61210); South Africa, on leaves of *V.karroo*, 15 May 2006, W. Maier WM3512 (PREM61220); South Africa, Western Cape, Worcester, on inflorescences of *V.karroo*, 20 December 2004, W. Maier WM3577 (KR-M-43406); South Africa, North-West Province 25°30'08.2"S; 27°21'32.4"E, on leaves of *V.karroo*, M. Ebinghaus ME433 (KR-M-43657).

##### Notes.

Morphological as well as phylogenetic analyses, based on nrITS and nrLSU sequence data of the specimens PREM61214, PREM61216, PREM61218, PREM61219, PREM61862 and PREM61875 collected from *V.natalitia* and *V.permixta*, respectively, supported conspecificity and their placement in *R.macowaniana*. Therefore, we report *V.natalitia* and *V.permixta* as new hosts for *R.macowaniana*.

#### Ravenelia evansii

Taxon classificationFungiPuccinialesRaveneliaceae

Sydow, Ann. Mycol. 10, p. 440, Monograph. Ured. 3, p. 234

Figure 4J

Ravenelia evansii Sydow, Ann. Mycol. 10, p. 440, Monograph. Ured. 3, p. 234 On Vachelliaborleae (Burtt Davy) Kyal. & Boatwr., V.davyi (N.E.Br.) Kyal. & Boatwr., V.exuvialis (I. Verd.) Kyal. & Boatwr., V.hebeclada (DC.) Kyal. & Boatwr., V.luederitziivar.retinens (Sim.) (JH Ross & Brenan) Kyal. & Boatwr. and V.swazica (Burtt Davy) Kyal. & Boatwr. (Fabaceae: Mimosoideae).

##### Emended description.

Telia subepidermally erumpent, dark brown to blackish, scattered or in loose groups on the abaxial side of leaflets, sori on the comparatively large leaflets of V.robustassp.robusta often forming concentric rings of 2.2–3.3 mm in diameter, single sori (120)230–500(710) μm in diameter with the largest telia appearing to develop in concentric arranged groups, subcircular to elongated; paraphyses lacking; teliospores circular to subcircular from topview, topside convex to almost hemispherical from lateral view, chestnut brown, (47)74–103(124) μm in diameter with (3)5–7(8) probasidial cells in a cross-section; single probasidial cells mostly single-layered, sometimes central cells and in rare events single cells two-layered, (16)23–30(39) μm from lateral view and (11)18–25(34) μm from top view; each probasidial cell with 3–5(8) spines; cysts hyaline and smooth, uniseriate and each cyst appears to be divided by a faint constriction, of the same number as peripheral probasidial cells, swelling in water but only slightly in lactophenol, pedicels compound, not persisting.

##### Specimens examined.

All specimens examined for the emended species description of *R.evansii* representing new host associations are given in Table 1.

##### Notes.

Rust infections on specimens of *Vachelliaborleae* (ME384, PREM61869), *V.davyi* (PREM61845, PREM61005), *V.exuvialis* (PREM61876, PREM61868), *V.hebeclada* (PREM61227), V.luederitziivar.retinens (PREM61846) and *Vachelliaswazica*.(PREM61002, PREM61028, PREM61008) were identified using morphological characters of the teliospores. These generally matched those of *R.evansii* Syd. & P. Syd. given in Doidge (1927) and Shivas et al. (2013) and were supported by molecular phylogenetic analyses of nrITS and nrLSU sequence data. These *Vachellia* species are reported as new hosts for *Raveneliaevansii*. Despite major congruence of teliospore characters, the range of the teliospore diameter observed by our examinations exceeded the size range of 50–80 μm and 63–90 μm given in Doidge (1927) and Shivas et al. (2013), respectively. Furthermore, the occurrence of two-layered probasidial cells is reported here for the first time Figure 4J). Therefore, we present an emended description of the telial stage of *R.evansii* Syd. & P. Syd. emend. Ebinghaus, W. Maier and Begerow.

**Table 3.** Summary of morphological characters and spore sizes of *Raveneliaevansii*, *R.macowaniana* and *R.xanthophloeae*. All size measurements are given in μm, minimum and maximum values in parentheses. ^†^Measurements and observations according to Doidge (1939).

	Teliospores			
	Diameter	Probasidial cell size	Ornamentation	No. of Cells in diameter
* R. evansii *	(47)70–95(124)	(16)24–29(40) × (12)18–25(34)	(1.5)4–6(8)	(3)5–7(8)
* R. macowaniana *	(50)82–105(118)	(19)23–32(40) × (13.5)18–24(34)	–	(3)5–6(7)
* R. xanthophloeae *	(40.5)65–75(82)	(19)22–25(39.5) × (12.5)19–23(32)	(0.5)1–2(3)	(3)4–5(6)
	**Aeciospores**	**Urediniospores**		
	**Size**	**Size**	**Germ pores**	
			**Number**	**Arrangement**
* R. evansii *	23–40 × 16–26^†^	25–35 × 18–24^†^	4^†^	equatorial^†^
* R. macowaniana *	24–35 × 17–28^†^	20–30 × 12.5–15^†^	4^†^	equatorial^†^
* R. xanthophloeae *	(19)21–24(29)×(14)17–19(23)	(18)23–26(38) × (13)16–20(25)	5–6	equatorial

## Discussion

The new rust species, *R.xanthophloeae*, was found only on the fever tree *Vachelliaxanthophloea*. In South Africa, this tree species is naturally confined to habitats with shallow watertables in low-altitude areas of the northeastern KwaZulu-Natal, Mpumalanga and Limpopo Provinces (Coates Palgrave 2005, Smit 2008). However, it is frequently planted as ornamental at higher altitudes throughout Southern Africa, where infections by *R.macowaniana* on *V.karroo* are common. Yet, despite extensive sampling efforts, no rust has been reported from *V.xanthophloea* in these regions, suggesting that *R.xanthophloeae* might currently be restricted to the native range of its host tree. Furthermore, *V.xanthophloea* is apparently resistant to infection by the frequently co-occurring and closely related *R.macowaniana*. This observation lends additional support to the separation of *R.macowaniana* and *R.xanthophloeae* as distinct species.

Sequence divergence was smaller amongst the specimens of *R.evansii* than within *R.macowaniana* (Fig. 1). This is in contrast to teliospore morphology, where the six examined teliospore traits showed considerable variability in *R.evansii*, but very little variation in *R.macowaniana*. Specifically, an effect of the host association on teliospore morphology could be demonstrated and this was most pronounced in specimens of *R.evansii* collected from *V.borleae*, *V.davyi* and *V.exuvialis*. It has been demonstrated in other fungal and oomycetous plant pathogens that infraspecific variation of spore traits might correlate with host species (Savile 1976, Lutz et al 2005, Runge and Thines 2011). However, mechanisms leading to such host-associated differences in rust fungi remain obscure. Savile (1976) hypothesised that differences in host compatibility of rust fungi potentially lead to differences in nutrient supply and could consequently influence morphological features. It was also speculated that host anatomy such as the thickness of the cuticle and epidermis might influence spore morphology (Scholler et al. 2011). Clearly, experimental studies that focus on the differential effects of the host and environment on morphological character expression in the rust fungi are needed to resolve this question.

In the present study, the host ranges of *R.macowaniana* and *R.evansii* were expanded from one to three and from four to nine hosts, respectively. Thus, these two rust species have a broader host range then previously reported and parasitise several co-occurring acacia species in the South African savannah biome. This is in contrast to recent findings in the genus *Endoraecium* that infect Australian wattles (*Acacia* s. str., formerly Acaciasubg.Phyllodineae). Based on morphological and molecular phylogenetic studies, species previously thought to have a broad host range were split into several species infecting mostly a single tree species (Berndt 2011, McTaggart et al. 2015). In this case, a general pattern of co-speciation of the parasites with their hosts was suggested (McTaggart et al. 2015). In the South African acacia rusts however, the shared distribution ranges of their hosts may prevent the rusts from speciation by recurrent gene flow between metapopulations. In contrast, *Raveneliaxanthophloeae* appears to infect only *V.xanthophloea*. In the phylogenetic analyses, this rust was closely related to *R.macowaniana*, which suggests a more recent speciation of both species. The host of *R.xanthophloeae* is eco-geographically clearly separated from the hosts of *R.macowaniana* and the different niches of the host most likely contributed considerably to the divergence of the parasite species.

## Supplementary Material

XML Treatment for Ravenelia xanthophloeae

XML Treatment for Ravenelia macowaniana

XML Treatment for Ravenelia evansii

## Supplementary materials

Supplementary material 1 Figure S1 Data type: statistical data Explanation note: Boxplot of measurements of the six defined teliospore characters of *R.evansii*. Values were obtained from teliospores derived from seven different host species of in total 18 individual trees. The boxplots are based on mean values calculated for all investigated teliospores for each specimen, respectively. This is an open access article distributed under the terms of the Creative Commons Attribution License (CC BY 4.0), which permits unrestricted use, distribution, and reproduction in any medium, provided the original author and source are credited.Malte Ebinghaus, Wolfgang Maier, Michael J. Wingfield, Dominik Begerow

Supplementary material 2 Figure S2 Data type: statistical data Explanation note: Boxplot of measurements of the six defined teliospore characteristics of *R.macowaniana* and *R.xanthophloeae*. Values were obtained from teliospores derived from three different host species of in total 10 individual trees. The boxplots are based on mean values calculated for all investigated teliospores for each specimen, respectively. This is an open access article distributed under the terms of the Creative Commons Attribution License (CC BY 4.0), which permits unrestricted use, distribution, and reproduction in any medium, provided the original author and source are credited.Malte Ebinghaus, Wolfgang Maier, Michael J. Wingfield, Dominik Begerow

Supplementary material 3 Table S1 Data type: species data Explanation note: List of measurements of teliospore characters of *R.evansii*. Obtained values were sorted by voucher and by individual teliospores. All measurements are given in μm. This is an open access article distributed under the terms of the Creative Commons Attribution License (CC BY 4.0), which permits unrestricted use, distribution, and reproduction in any medium, provided the original author and source are credited.Malte Ebinghaus, Wolfgang Maier, Michael J. Wingfield, Dominik Begerow

Supplementary material 4 Table S2 Data type: species data Explanation note: List of measurements of teliospore characters of *R.macowaniana* and *R.xanthophloeae*. Obtained values were sorted by voucher and by individual teliospores. All measurements are given in μm. This is an open access article distributed under the terms of the Creative Commons Attribution License (CC BY 4.0), which permits unrestricted use, distribution, and reproduction in any medium, provided the original author and source are credited.Malte Ebinghaus, Wolfgang Maier, Michael J. Wingfield, Dominik Begerow
